# Effectiveness of Open Rigid Internal Fixation of Condylar Fracture Resulting in Temporomandibular Joint Function Recovery

**DOI:** 10.3390/dj13120562

**Published:** 2025-12-01

**Authors:** Paulina Agier, Szymon Tyszkiewicz, Marcin Kozakiewicz

**Affiliations:** 1 Multispecialty Dental Clinic 106/116 Kościuszki Av., 90-442 Lodz, Poland; paulinaagier2402@gmail.com; 2 Department of Maxillofacial Surgery, Medical University of Lodz, 251 Pomorska Str., 92-213 Lodz, Poland; szymon.tyszkiewicz@umed.lodz.pl

**Keywords:** condyle, mandible, open rigid internal fixation, surgery, temporomandibular joint, Helkimo Index, complication, rehabilitation

## Abstract

**Background**: Maxillofacial trauma can impair crucial functions of the stomatognathic system. Mandibular condyle fractures, in particular, often lead to temporomandibular joint (TMJ) dysfunction. **Methods**: This study evaluated the effectiveness of open rigid internal fixation (ORIF) in restoring TMJ function, using the Helkimo Index to compare pre- and post-operative outcomes. **Results**: A total of 395 patients who underwent ORIF for condylar fractures were analyzed (302 males, 93 females). TMJ function improved significantly from baseline to 6-month follow-up (*p* < 0.001), with a mean reduction of 2.18 grades on the Helkimo Index. Higher post-operative Helkimo grades (2–3) occurred more frequently during warm months than during cold months (*p* < 0.05). Low body mass index (BMI) was associated with a greater risk of post-surgical TMJ dysfunction (*p* < 0.001). TMJ function correlated with facial nerve recovery: patients with poorer pre-operative TMJ function showed additionally slower facial nerve recovery during the first five months after surgery. Age, gender, place of residence, injury characteristics, comorbidities, delay of surgery, duration of surgery, surgical approach, fixing material and laboratory blood tests showed no significant association with post-operative TMJ function. Residual TMJ dysfunction was observed in 3% of treated patients (Di = 3). **Conclusions**: ORIF, combined with appropriate post-operative physiotherapy, effectively restores TMJ function after condylar fractures—including severe injuries. Simple clinical indices such as the Helkimo Index reliably capture functional improvement.

## 1. Introduction

Nowadays, when medicine is becoming increasingly advanced, the treatment of bone fractures must meet several essential requirements: restoration of bone continuity and stability [1], preservation of proper joint [2], muscle [3,4,5,6], and nerve function [7], achievement of satisfactory esthetic outcomes with minimal or no scarring [8], and establishment of conditions that promote complete healing [9]. In maxillofacial surgery, meeting all these requirements is particularly challenging in many cases [10]. Among maxillofacial injuries, mandibular fractures are the most common [11,12,13]. Fractures of the mandible directly affect many functions of the facial region, and condylar fractures are considered particularly difficult to manage due to their complex anatomical and functional implications [14].

The treatment of condylar fractures should be based on minimally invasive and precise techniques to minimize the risk of facial nerve injury [15,16], ensure proper occlusion [17], prevent unaesthetic salivary fistulas [18], and minimize visible scarring [19]. In selected cases, endoscopic approaches may be applied [20,21,22]. Although esthetic outcomes are important, surgeons must also prioritize the restoration of functional integrity of the stomatognathic system [23].

The condylar region presents specific surgical challenges due to its small size and difficult anatomical location [24,25]. The condylar head forms an integral structural and functional component of the temporomandibular joint (TMJ) [26]. The TMJ is covered by a different type of cartilage tissue—fibrocartilage—whereas most joints in the musculoskeletal system are covered by hyaline cartilage [27]. Fibrocartilage is characterized by high resistance to both compression and tensile forces [28,29,30]. This type of cartilage is also found in the intervertebral disks, which are known to be well adapted to withstand heavy loads and pressure [31,32]. The TMJ is also a highly specialized joint in the human body; movement of one TMJ inevitably induces movement in the contralateral joint, as both are connected through the mandible [33,34]. Consequently, impairment of one TMJ leads to compensatory overloading of the other, which increases the risk of secondary dysfunction.

The functional significance of the TMJ is considerable, as it enables fundamental life activities [33] such as chewing, speaking, swallowing, and even partially breathing. Therefore, post-operative care should include systematic assessment and monitoring of TMJ function, as overlooking complications [35,36] may lead to serious TMJ disorders such as joint ankylosis or mandibular head resorption [37,38]. Disturbed TMJ function in many patients manifests as trismus and/or muscle and facial pain. Clicking and crackling sounds in the temporomandibular joint are also frequently observed [39,40,41]. TMJ disorders may additionally affect skeletal conditions [42], interfering with posture, respiratory function, or even pelvic alignment [43]. Altered muscle–fascia chain tension may, therefore, influence the entire body.

The useful tool to perform TMJ examination is the Helkimo Index, which is a widely used, reliable, and easy-to-perform clinical assessment system designed to evaluate temporomandibular joint (TMJ) function, compare pre- and post-operative outcomes, and maintain standardization in clinical documentation. The index, originally developed by Helkimo in 1974 [44] to provide a comprehensive evaluation of the stomatognathic system, is one of the most commonly applied instruments in dentistry, maxillofacial surgery, and stomatognathic rehabilitation.

The original Helkimo Index consists of three components: the Anamnestic Index (Ai), which records subjective symptoms reported by patients; the Occlusal Index (Oi), which evaluates occlusal disturbances; and the Clinical Dysfunction Index (Di), which clinically assesses TMJ function across five domains—range of mandibular movement, joint function, muscle pain on palpation, joint pain on palpation, and pain during mandibular movement. Among these components, the Clinical Dysfunction Index (Di) has become the most widely used in contemporary research and clinical practice, particularly in maxillofacial surgery and TMJ rehabilitation.

The aim of this study was to evaluate the effectiveness of ORIF combined with physiotherapy to restore TMJ function after mandibular condylar fracture and to identify potential risk factors influencing post-operative functional outcomes.

## 2. Materials and Methods

Medical records were reviewed to extract retrospective descriptive data on patients treated for mandibular condylar process fractures between 2017 and 2024. Patients were identified in the hospital medical database, and all data were anonymized prior to analysis. Institutional approval was obtained before data collection. The study design followed the Strengthening the Reporting of Observational Studies in Epidemiology (STROBE) guidelines [45]. As the study was retrospective, the sample size was determined by the number of patients meeting all inclusion criteria.

The inclusion criteria were: a confirmed diagnosis of mandibular condylar process fracture, treatment with ORIF, and attendance at 6-month follow-up appointments. The exclusion criteria were: closed treatment, incomplete medical history, missing follow-up appointments, bruxism in the pre-injury medical history, pre-injury TMJ dysfunction, history of head and neck cancer, metabolic bone disease, and a history of orthognathic surgery.

**Figure 1:**
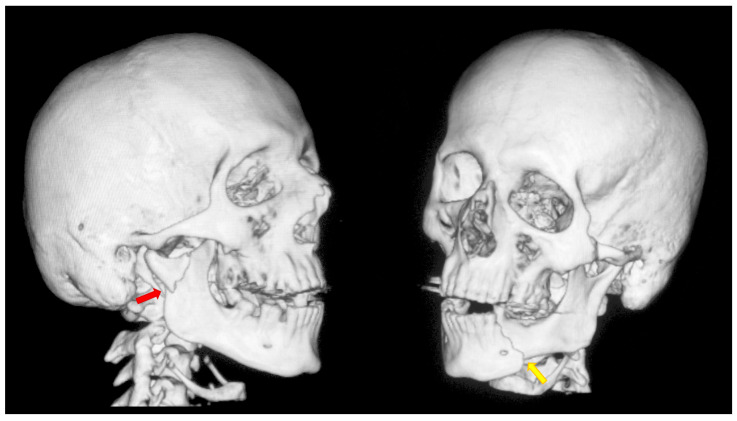
A typical case of mandibular injury. Computed tomography scan performed prior to treatment. The red arrow indicates a fracture at the base of the mandibular condyle. The yellow arrow indicates an additional fracture in the contralateral mandibular body. This is an epidemiologically typical situation [51]. A fracture of the mandibular condyle very frequently occurs together with another fracture of the mandible. The coexistence of two fracture lines causes significant instability of bone fragments and causes typical malocclusion.

**Figure 2:**
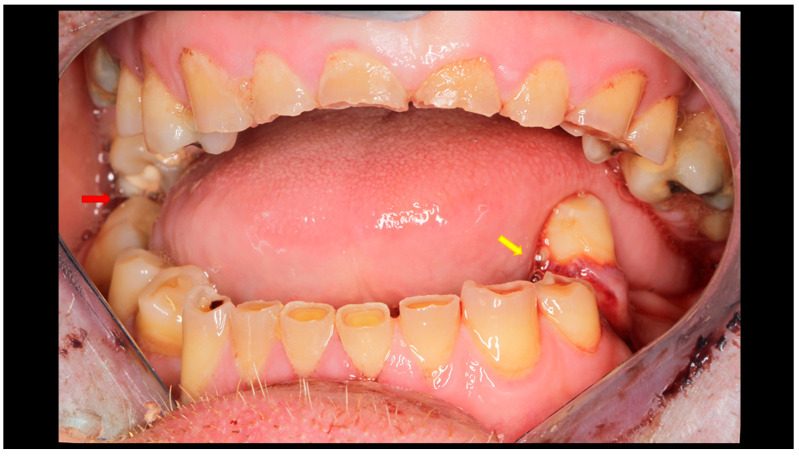
Post-traumatic malocclusion. The mandible is divided into three pathological parts. The proximal fragments are displaced upward, while the distal fragment (the middle one) is displaced downward. This results in a completely open bite with dental contact only on the last teeth in the dental arch (red arrow). Please also note the break in the occlusal line resulting from an open fracture of the mandible on the left side (yellow arrow).

After physical and radiological examination, mandibular condylar process fractures were classified into six diagnostic categories: basal, low-neck, high-neck, and head fractures (types A, B, or C) [46,47,48]. The severity of the injury was determined according to the AO CMF classification system, level 3 [49] (Figure 1). Temporomandibular joint (TMJ) function was assessed using the Helkimo Clinical Dysfunction Index (Di) (from 0: no dysfunction to 3: severe dysfunction) (Table 1) [44] (Figure 2), and facial nerve function was evaluated according to the six-grade House–Brackmann scale (from 1: normal nerve function to 6: total loss of function) [50].

**Figure 3:**
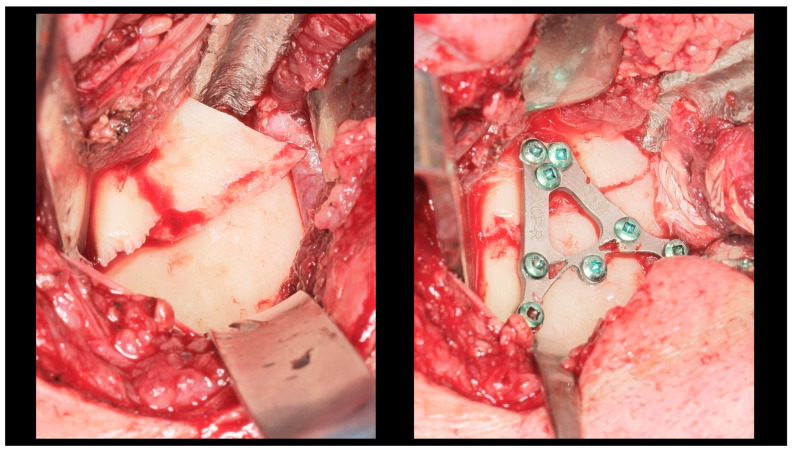
Intra-operative view (retromandibular transparotid approach). The most commonly observed finding is lateral displacement with overlapping bone fragments (see **left** side). Osteosynthesis was performed using an XCP plate designed for fixation of the right side (see **right** side).

**Figure 4:**
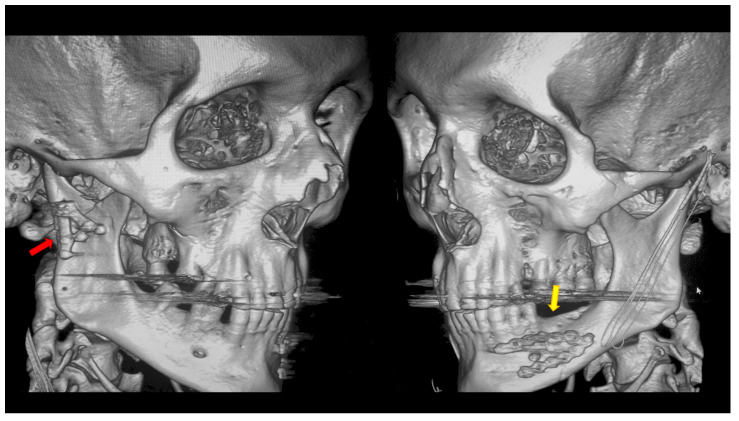
Open rigid internal fixation was performed using a dedicated plate in the right condylar process (red arrow) and two straight plates of the 2.0 system on the left side of the mandibular body (yellow arrow).

**Figure 5:**
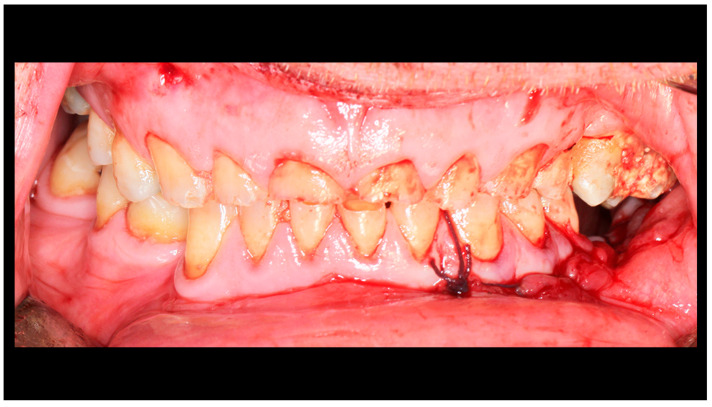
Occlusion immediately after fixation (intraoperative photo). Pre-trauma occlusal conditions have been restored.

All patients underwent ORIF under general anesthesia with nasal intubation. Pre-operative antibiotic prophylaxis was administered in cases of concomitant open fractures. The surgical approach selected for operative management was closely related to the location of the fracture, particularly the height of the fracture. Basal fractures were treated using several approaches, including the retromandibular, periangular, and intraoral endoscopic-assisted approach, as well as variants of classic approaches. Low-neck fractures were most often managed using a preauricular approach extended downward, whereas high-neck fractures were typically treated using a preauricular approach extended upward. Condylar head fractures, known to be the most challenging to access and reduce, were managed using approaches such as the auricular, preauricular, and retroauricular approaches. In all cases, the final choice of approach depended on the specific characteristics of the fracture. ORIF was achieved using different types of fixation materials. In our clinic, ACP plates, XCP plates, straight plates, and compressive screws were used (ChM, Juchnowiec Kościelny, Poland; www.chm.eu, accessed on 13 November 2025). The crucial objective of open surgical treatment for condylar fractures was to achieve accurate anatomical reduction and stable rigid fixation. In all cases, the surgeons aimed to minimize the interfragmentary gap as much as possible, maintaining it at a hairline width. Achieving such close adaptation of the bone fragments was considered a fundamental condition for optimal fracture healing (Figure 3, Figure 4 and Figure 5).

**Figure 6:**
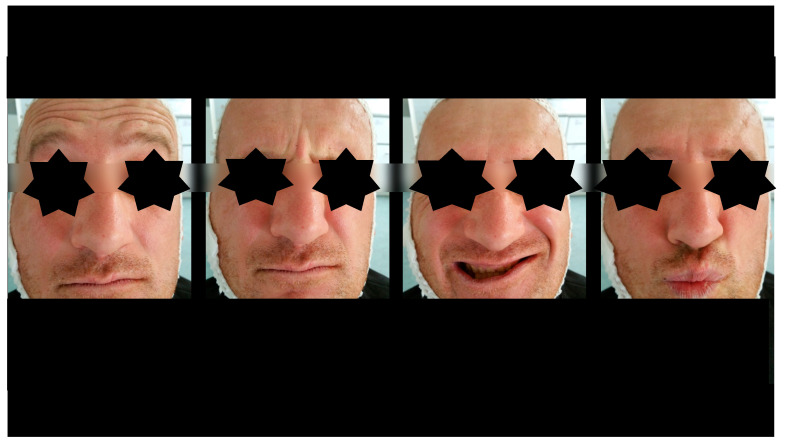
Facial expressions two days after surgery. Weakness of the mandibular marginal branch of the left facial nerve. This is related to fixation of the mandibular body.

**Figure 7:**
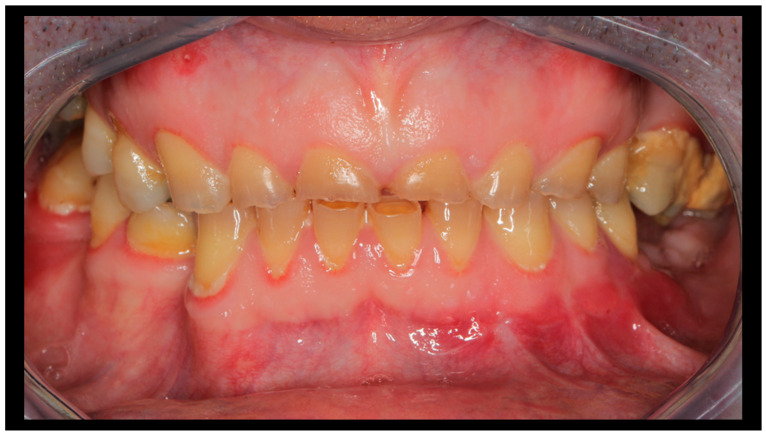
Occlusal conditions 4 weeks after completion of treatment.

During hospitalization, facial nerve function was assessed and wound care was provided (Figure 6). Skin sutures were removed after 7–10 days. At follow-up visits, facial nerve and TMJ function, as well as possible complications, were examined, and patients with complications received appropriate treatment (Figure 7). At six-month follow-up after surgery, radiological control was performed.

**Figure 8:**
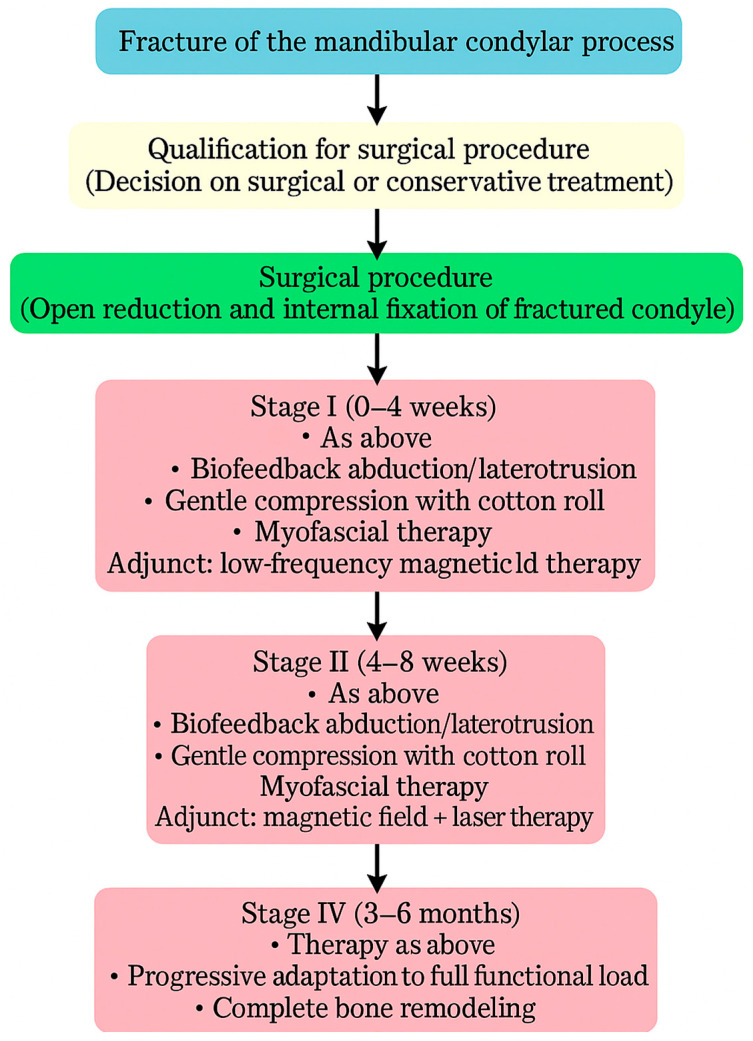
Flowchart illustrating the treatment–rehabilitation process after condylar fracture. During the first stage of rehabilitation, low-frequency magnetic field therapy was applied in two-week cycles of stimulation alternating with two weeks of rest. During the second stage of rehabilitation, low-frequency magnetic field therapy combined with low-level laser therapy was applied in two-week cycles of stimulation alternating with two weeks of rest, initiated after the onset of neovascularization and regeneration of the periosteum.

**Figure 9:**
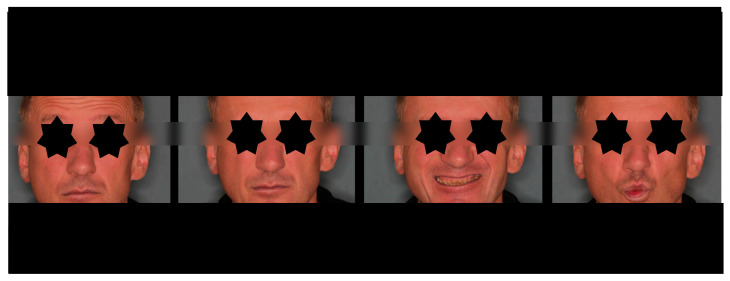
Facial expressions after physiotherapy treatment. Full function was restored within 4 weeks after treatment.

Physiotherapy was implemented to restore the full function of the stomatognathic system (Figure 8). Post-operative physiotherapy was divided into four phases (Figure 9). In the first phase, the primary therapeutic objectives were to prevent akinesia and to reduce edema that could impair local microcirculation [53] or exert compressive effects on peripheral nerve branches. To achieve these goals, rehabilitation included lymphatic drainage, facial muscle exercises, isometric exercises, and active movements performed within a pain-free range [54]. The second phase expanded the therapy with manual techniques and biofeedback training, supporting proper tissue healing by preventing intertissue adhesions and restrictive scar formation. In both early phases, physical modalities were used to facilitate tissue remodeling and the restoration of functional fiber integrity. Low-frequency magnetic field therapy was applied from the first phase (acute stage: 1–5 Hz, 0.5–3 mT; subacute stage: 5–20 Hz, 3–5 mT; rectangular pulse; 30–45 min), with gradually increasing impulse strength every 2–3 sessions. Low-level laser therapy was introduced in the second phase to enhance tissue regeneration and microcirculation (100–400 mW, fwavelength 635 nm, 1–4 J/cm^2^, applied using a sweeping technique). Both modalities were administered in two-week stimulation cycles alternating with two-week breaks to prevent habituation to the therapeutic stimulus. The third phase of physiotherapy aimed to restore full mandibular range of motion, recover the function of the affected peripheral nerve branches, and evaluate both the quantitative and qualitative characteristics of TMJ intra-articular mechanics [55]. In addition to the previously introduced manual therapy and home exercise programs, this phase incorporated exercises designed to maximize the expansion of free mandibular movements, targeted manual mobilization of muscles and joints, and both concentric and eccentric strengthening exercises. The fourth phase represented a continuation and progression of the third-phase therapeutic objectives, focusing primarily on achieving full, functional TMJ mobility and restoring normal loading capacity in accordance with the biomechanical tolerance and remodeling capacity of the healing tissues.

Medical data were categorized into five groups: demographic and anthropometric data (age, gender, body mass index [BMI], place of residence); injury characteristics (injury reason, use of intoxicants, fracture diagnosis, number of mandibular fractures, month of injury); treatment characteristics (initial place of treatment, delay to surgery, duration of surgery, surgical approach, fixation material); comorbidities and laboratory findings (comorbidities, pre-operative hemoglobin level [HGB], pre-operative white blood cell count [WBC]); and post-operative outcomes and complications (TMJ function, periauricular skin desensitization, facial nerve function, salivary fistula, intraoperative facial nerve neurotmesis).

The statistical analysis was performed using Statgraphics Centurion 18 (Statgraphics Technologies, Inc., The Plains, Warrenton, VA, USA; www.statgraphics.com, accessed on 23 September 2025). The analysis was based on data from 395 patients. Descriptive statistics were used to summarize the distribution and central tendency of quantitative variables. The non-parametric Kruskal–Wallis test, followed by Bonferroni-adjusted post hoc comparisons, was used to assess differences in medians among subgroups. Changes in the Helkimo Dysfunction Index before and after treatment were assessed using the sign test for paired samples, testing the hypothesis of median equality. Associations between categorical variables and treatment outcomes were analyzed using the Chi-square test of independence. To evaluate the impact of clinical factors on Helkimo Dysfunction Index values before and after surgery, simple linear regression analyses were performed to assess the associations between the Helkimo Dysfunction Index and the House–Brackmann scale at different time points. A *p*-value < 0.05 was considered statistically significant. Missing data were not imputed; all analyses were performed using a complete-case approach. To assess whether missing data could have influenced the reliability of statistical inference, a priori sample size estimations and post hoc power analyses were performed for all main statistical procedures applied in the study. These included the one-way ANOVA (and the Kruskal–Wallis test for non-normally distributed continuous variables), the Chi-square test (and Fisher’s exact test for categorical variables with low expected counts), and correlation or simple linear regression analyses (Pearson’s or Spearman’s, as appropriate) [56,57,58].

## 3. Results

**Figure 10:**
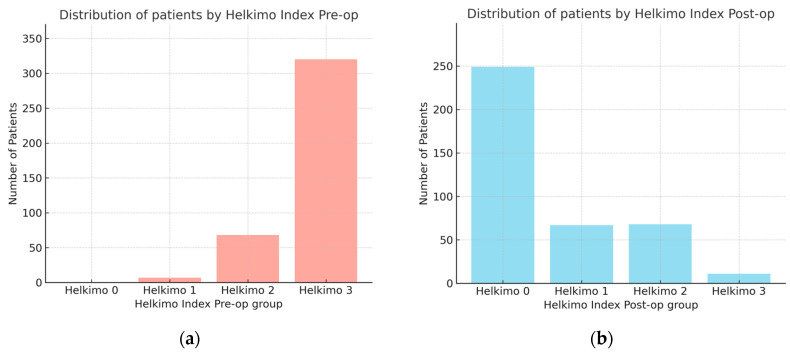
Comparison of number of patients with two groups assessed using Helkimo Clinical Dysfunction Index measured pre-operatively (**a**) and post-operatively (**b**). Significant differences are observed in the distribution of Helkimo Index grades between the two groups. The significant improvement in TMJ function is demonstrated (*p* < 0.001). Abbreviations: Pre-op—pre-operatively; Post-op—operatively.

The observational study analyzed medical records of 395 patients who underwent surgical treatment for mandibular condylar process fractures. In this cohort, the male-to-female ratio was 302:93. Male patients were significantly younger (mean age: 38 ± 15 years) compared with female patients (mean age: 46 ± 20 years), as determined by the Kruskal–Wallis test. The comparison of Helkimo Di measured pre-operatively (pre-op) and 6 months post-operatively (post-op) showed significant differences in TMJ function before and after surgical treatment with rehabilitation. Pre-op scores were distributed as follows: Di 0—0%, Di 1—1.8%, Di 2—17.2%, and Di 3—81%. At the 6-month follow-up, the distribution had shifted markedly: Di 0—63%, Di 1—17%, Di 2—17%, and Di 3—3% (Figure 10a,b). The mean pre-op score was 2.79 ± 0.45 (median 3.0; 95% CI: 2.75–2.84), whereas the post-op score decreased to 0.61 ± 0.89 (median 0; 95% CI: 0.52–0.71). The mean difference was 2.18 (95% CI: 2.08–2.28), confirming a statistically and clinically significant improvement in TMJ function and demonstrating the high effectiveness of surgical treatment of mandibular condylar fractures.

**Figure 11:**
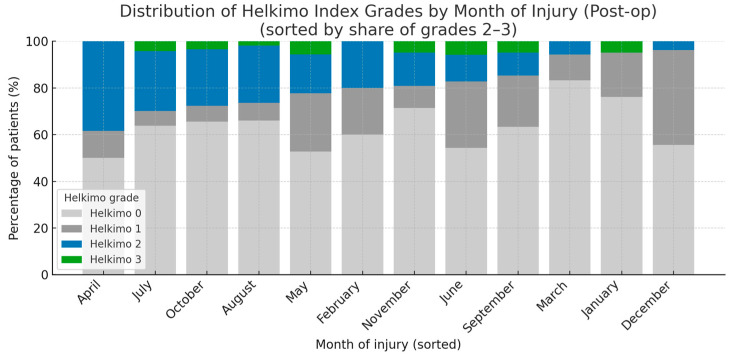
The figure presents the distribution of all the Helkimo Index grades among the examined patients, showing the frequency of each grade in every month of the year. The months are ordered according to the proportion of grades 2–3. The vertical axis indicates the percentage of patients, while the horizontal axis represents the months of the year. A statistically significant association was observed (*p* < 0.05). Abbreviations: Post-op—post-operatively.

A statistical analysis of five main categories of potential risk factors influencing post-op TMJ function was performed: demographic and anthropometric data (age, gender, BMI, place of residence); injury characteristics (injury reason, intoxicants, fracture diagnosis, number of injured condyles, number of mandible fractures, month of injury); treatment characteristics (initial place of treatment, delay of surgery, duration of surgery, surgical approach, fixation material in detail); comorbidities and laboratory blood tests (comorbidities, pre-op HGB, pre-op WBC count); and post-op outcomes (salivary fistula, periauricular skin desensitization, facial nerve intraoperative neurotmesis) (Table A1). In total, 23 factors were examined, but only two reached statistical significance: BMI and month of injury, while the remaining factors did not. Patients categorized as grades 2–3 according to the Helkimo Di had lower BMI values than those categorized as grades 0 or 1 (*p* < 0.001). Moreover, a higher proportion of patients with post-op Helkimo Di grades 2 or 3 was observed among those who sustained injuries during the spring–summer period (*p* < 0.05). Notably, grade 3 was recorded only in seven months of the year, namely May, June, July, August, September, October, and November. A markedly lower number of patients presenting with Post-op significant signs of TMJ dysfunction (Helkimo Di grades 2–3) was observed 6 months after surgery among those who sustained injuries in the winter months, specifically December, January, and March (Figure 11).

To examine the potential influence of facial nerve function on TMJ function, logistic regression analysis was performed. The evaluation compared House–Brackmann scale scores during the 6-month follow-up with both pre-op and post-op Helkimo Index scores. The correlation between post-op facial nerve function and post-op Helkimo Index scores did not reach statistical significance in any of the follow-up months. Therefore, no association was found between post-op facial nerve function, assessed using the House–Brackmann scale, and TMJ function, evaluated according to the Helkimo Index.

The situation differed when post-op facial nerve function was correlated with pre-op Helkimo Index scores. In our study, patients with higher House–Brackmann scale grades were more likely to have previously presented with higher pre-op Helkimo Index scores after injury. The strength of this association showed an increasing tendency from the first to the second month, with the lowest *p*-value observed in the second month (02M, *p* < 0.01), indicating the strongest statistical significance during the entire follow-up. In the third month (03M, *p* < 0.01), the correlation still had high statistical significance, confirming the relationship between impaired facial nerve function and more severe pre-op TMJ dysfunction (Table 2). However, from that point onward, a gradual decline in the correlation was observed. By the sixth month of follow-up, the statistical significance had disappeared (*p* = 0.151), suggesting that the initial relationship between facial nerve impairment and pre-op TMJ dysfunction diminished over time, most likely as a result of healing and rehabilitation processes affecting both the TMJ and the facial nerve.

Analyzing the statistical data allows for a more detailed characterization of the Di 3 grade post-operative group (2). The total number of patients was 12 (8 males and 4 females). The most frequent cause of injury was assault (4 cases). The predominant type of fracture was a basal fracture (6 cases). In most patients, bilateral fractures were observed (10 cases). The mean delay from injury to surgery was 24 days (compared with 9 days in the total cohort), representing the longest delay among all four Helkimo post-operative grades (Di 0, Di 1, Di 2, and Di 3). The mean duration of surgery was also the longest among all analyzed groups, with an average of 204 min (compared with 191 min in the total cohort). The most commonly used fixation material was compressive screws (5 cases). It is important to highlight that these data did not reach statistical significance (Table A1); however, they may serve as valuable clinical reference points, facilitate inter-center comparisons, and provide a basis for future research discussions.

## 4. Discussion

TMJ dysfunction significantly impacts patients’ quality of life. Therefore, it is crucial to provide appropriate surgical treatment and post-operative rehabilitation, particularly in cases of mandibular fractures, and especially fractures of the mandibular condyle, which constitute a significant proportion of maxillofacial trauma [51,59,60,61,62]. This condition is particularly common among young males [51,59,60,61,62]. Although young males are more frequently affected in condylar traumatology, in our study neither gender nor age influenced the healing process or post-operative TMJ function. These findings are consistent with reports from several other studies examining TMJ disorders [63,64,65]. In contrast, research conducted by Leuin et al., who examined a population of children and young adults, indicated that female gender was a risk factor for post-traumatic TMJ disorders [66].

Current scientific literature widely supports the thesis that surgical treatment of mandibular condyle fractures provides good functional outcomes [67,68,69]. This is reflected in the significant improvement of TMJ function observed pre-operatively and post-operatively, as also demonstrated in our study. Such outcomes are achieved through proper restoration of bony continuity and rigid fixation of the fracture [70], allowing immediate recovery of TMJ anatomy after surgery. To ensure optimal results, not only successful surgery but also appropriate post-operative physiotherapy is recommended [71,72,73].

Despite the advantages of ORIF, the surgical procedure itself may cause tissue damage, including disruption of the skin, mucosa, deeper soft tissues, or periosteum, which can prolong bone healing by compromising one of its key vascular sources [74,75]. Moreover, surgery carries the risk of infection and inflammation, further disturbing repair. To minimize these risks, the healing process must be carefully managed [76]. Early mobilization and self-therapy exercises help prevent local akinesia and lymphatic accumulation, reducing the likelihood of fibrosis and tissue stiffening. Controlled compression–traction stimulation at the fracture site, in line with Delpech–Wolff’s law, together with prevention of tissue overloading according to the Arndt–Schulz principle, are essential for optimal recovery [77]. Finally, rehabilitation should aim to prevent adhesions, scars, and abnormal neuronal activity that may result in hypersensitivity, neuropathy, or neuralgia, including trigger points [78].

Therapeutic success in the management of mandibular condylar fractures can be achieved only when both TMJ and facial nerve function are fully restored. In our study, there appeared to be a correlation between the pre-operative Helkimo Index grade and the post-operative recovery of facial nerve function. This suggests that the more severe the initial injury, the higher the likelihood of visible post-operative facial nerve dysfunction. This observation seems to be in line with previously published reports. Pienkohs et al. [79] demonstrated that greater bone fragmentation is associated with longer surgical procedures. Other authors have indicated that prolonged procedures increase the risk of post-operative facial nerve palsy [15,80]. Moreover, more complicated fractures that require extended operative time necessitate wider tissue dissection, which in turn provokes traction and mechanical stress on soft tissues [81]. In addition, Cheng et al. [82] identified prolonged operative time as a significant risk factor for post-operative wound infection. Taken together, these findings may help explain the correlation between the pre-operative skeletal condition and the functional outcome of the facial nerve after surgery.

Proper nutrition plays a fundamental role in the healing of both soft tissues and bone. An adequate intake of proteins and essential minerals is crucial for creating optimal conditions for recovery [83,84,85]. One of the parameters commonly used to evaluate overall nutritional status is BMI [86]. Evidence in the medical literature indicates that lower BMI values, often reflecting insufficient nutritional intake, are associated with delayed or impaired healing processes and may increase the risk of post-operative complications [18,87,88,89,90,91]. For example, Demling demonstrated that adequate nutritional support is a critical component of the healing process, whereas protein–energy malnutrition can lead to serious complications and significantly hinder tissue repair [92]. The findings reported by Karpouzos et al. indicate that inadequate intake of essential vitamins, nutrients, and minerals can adversely affect bone healing throughout the post-fracture recovery period [93]. The research conducted by Stechmiller demonstrated that nutritional deficiencies may be associated with reduced wound tensile strength and an increased incidence of infection [94]. Numerous studies have shown that low BMI may coexist with impaired muscle strength and reduced physical performance [95,96]. Moreover in more severe cases, a lower BMI can also be associated with the development of sarcopenia [97,98]. Our observations are consistent with numerous scientific reports indicating that lower BMI is associated with less favorable recovery—in our case, the recovery of TMJ function. Similar results were documented in another study conducted on the same patient cohort from the same clinic, where low BMI values were linked to a longer duration of salivary fistula treatment when this complication occurred [18].

Summer is typically associated with a higher incidence of maxillofacial trauma [99,100]. During this period, injuries from bicycle [101], e-scooter [102], and sports accidents [103,104] occur more frequently, particularly among children and young adults [100]. The study conducted by Davis and Denaley, which compared bicycle-related injuries during the winter and summer seasons, indicated a significantly higher incidence of injuries in summer, reaching an 8:1 ratio of summer to winter cases [105]. Blomberg et al., in their study examining electric scooter–related injuries, also demonstrated a clear peak in injury incidence during the summer months compared with the winter period [106]. Moreover, the findings of a 16-year analysis of equestrian injuries by Meredith et al. also demonstrated that the incidence of injuries was significantly higher during the summer months compared with the winter period [107]. The findings of Vongsachang et al. are in line with previous research, as the authors demonstrated that every 10 °C increase in outside temperature was associated with a 24% increase in the probability that a fall would be injurious. Moreover, the ratio of injurious summer falls to winter falls clearly indicated that the summer season is more strongly associated with injury risk [108]. In our cohort, the greater number of cases with post-operative TMJ impairment recorded during the warmer months may reflect both the overall increase in accidents and a shift toward more severe injuries in summer. This is likely reflected in poorer post-operative Helkimo Index outcomes. In addition, environmental factors specific to summer—such as elevated ambient temperatures—may negatively affect the healing process [109]. Heat exposure has been associated with greater tissue edema and prolonged inflammatory responses, potentially contributing to delayed wound healing and greater discomfort [110]. These factors may impair functional recovery and partly explain the worse clinical outcomes observed in this group.

This study has several important limitations. First, the examined period included the COVID-19 pandemic, during which hospital regimens differed markedly from standard practice, treatment and rehabilitation protocols were modified due to governmental restrictions, and the overall attitude of many patients towards treatment was different from usual [111,112,113,114,115,116]. Second, the retrospective design of this study was associated with incomplete medical records and variability in patient examinations, surgical procedures, and medical history documentation, which were performed by different physicians over a 7-year period. This resulted in missing data that may have influenced the statistical outcomes and introduced a potential bias in data collection. The sample size was determined by the availability of medical records. Finally, this was a single-center study based on one population from a single country, which may limit the generalizability of the findings.

Further research is warranted to replicate and expand these findings in larger and more diverse cohorts, as the current evidence may not fully reflect the variability across different patient populations. Future investigations should particularly aim to include participants with varied demographic and clinical backgrounds to strengthen the external validity of the results. The authors also encourage future researchers to examine vitamin D levels both pre- and post-operatively. Moreover, multi-institutional collaborations are strongly recommended, as they would provide broader representation, minimize the risk of institutional bias, and enhance the generalizability of the conclusions to wider clinical settings.

## 5. Conclusions

Although maxillofacial trauma is common and may lead to significant dysfunction of the stomatognathic system, such as TMJ impairment caused by mandibular condylar process fractures, proper ORIF combined with adequate post-operative physiotherapy can restore TMJ function and re-establish overall stomatognathic system performance, even in severe cases. Functional outcomes of the TMJ can be reliably assessed using the Helkimo Index, allowing for direct comparison of pre- and post-treatment results.

## Figures and Tables

**Table 1:** The Clinical Dysfunction Index (Di) assesses five domains of temporomandibular joint function, each scored as 0, 1, or 5 points. The total score (0–25) defines the severity of dysfunction, with Maximum Interincisal Opening (MIO) ≥ 40 mm and lateral movements ≥ 7 mm considered normal. The Anamnestic Dysfunction Index (Ai) and the Occlusal Index (Oi) were not applied in this study [44,52].

Domain	Score 0	Score 1	Score 5
**A. Range of mandibular movement**	Normal opening and excursions (MIO ≥ 40 mm, lateral movements ≥ 7 mm)	Slightly reduced opening or lateral movement	Severely restricted mandibular movements
**B. TMJ function (movement)**	Smooth, symmetrical movements	Deviation or clicking during opening/closing	Locking, severe deviation, or crepitus
**C. Muscle pain on palpation**	No pain	Mild tenderness at 1–2 muscle sites	Severe pain in ≥3 muscle sites
**D. TMJ pain on palpation**	No pain	Mild unilateral TMJ pain	Severe or bilateral TMJ pain
**E. Pain during mandibular movement**	No pain	Mild pain during opening/closing	Severe pain during movement
**Interpretation of Total Score**			
Total Scores	Gradual interpretation	Clinical interpretation	
0	Di 0	No dysfunction	
1–4	Di 1	Mild dysfunction	
5–9	Di 2	Moderate dysfunction	
10–25	Di 3	Severe dysfunction	

Abbreviations: TMJ—temporomandibular joint; Di—dysfunction index.

**Table 2:** The table presents the correlation coefficients and corresponding *p*-values for the association between pre-op Helkimo Index and post-op facial nerve function assessed during follow-up visits.

Month Follow-Up	Correlation Coefficient	*p*-Value
00M	0.091	0.077
01M	0.143	**0.007**
02M	−0.171	**0.002**
03M	−0.159	**0.004**
04M	−0.137	**0.014**
05M	0.119	**0.035**
06M	0.082	0.151

Abbreviations: 00M—immediately post-op; 01M, 02M, 03M, 04M, 05M, 06M—1, 2, 3, 4, 5, 6 months post-op. Statistically, significant values (*p* < 0.05) are indicated in bold.

## Data Availability

The data on which this study is based will be made available upon request at www.researchgate.net/profile/Marcin-Kozakiewicz.

## Abbreviations

The following abbreviations are used in this manuscript:

ORIF	Open Rigid Internal Fixation
TMJ	Temporomandibular joint
Di	Clinical dysfunction index
Ai	Anamnestic dysfunction index
Oi	Occlusal dysfunction index
BMI	Body mass index
CHF	Condylar head fracture
MIO	Maximum interincisal opening
HGB	Hemoglobin level
WBC	White blood cell
ACP	‘’A’’—shape condylar plate
XCP	‘’X’’—shape condylar plate
M	Month

## Appendix A

**Table A1:** The original statistic data evaluation for potential factors influencing Post-op TMJ function—measured in Helkimo Dysfunction grades (0–3).

Variable	Variants	Available N	H0 (Mean ± SD/%)	H1 (Mean ± SD/%)	H2 (Mean ± SD/%)	H3 (Mean ± SD/%)	*p*-Value
Age [years]	Full cohort	395	39.89 ± 17.36	41.08 ± 16.04	40.39 ± 14.97	40.75 ± 11.44	*p* = 0.796
Gender	Female	395	12.91%	4.56%	6.08%	1.01%	*p* = 0.082
	Male		49.87%	12.41%	11.14%	2.03%	
BMI [kg/m^2^]	Full cohort	382	**23.12 ± 4.25**	**24.60 ± 4.18**	**21.54 ± 3.88**	**23.22 ± 3.75**	***p*** **< 0.001**
Residence place	Rural	395	19.24%	3.54%	4.30%	1.77%	*p* = 0.161
	Urban		43.54%	13.16%	13.16%	1.52%	
Injury reason	Assault	359	20.89%	4.46%	6.96%	1.11%	*p* = 0.409
	Fall		24.79%	6.69%	6.96%	0.84%	
	Sports		1.11%	0.28%	0%	0%	
	Vehicle		13.65%	4.46%	3.62%	0.84%	
	Workplace		2.23%	0.84%	0%	0.51%	
Intoxicants	No	358	36.31%	10.34%	9.22%	2.51%	*p* = 0.325
	Yes		26.54%	6.42%	8.10%	0.56%	
Fracture diagnosis	CHF A	359	0.56%	0%	0%	0%	*p* = 0.671
	CHF B		6.13%	0.84%	0.56%	0%	
	CHF C		16.43%	5.57%	5.57%	1.11%	
	High—neck		1.39%	0.84%	1.11%	0.28%	
	Low—neck		6.41%	1.11%	2.23%	0.28%	
	Basal		31.75%	8.36%	7.80%	1.67%	
Number of injured condyles	Unilateral	359	19.78%	5.01%	6.69%	0.56%	*p* = 0.439
	Bilateral		42.90%	11.70%	10.58%	2.79%	
Number of mandible fractures	1 Fracture	359	20.06%	5.29%	4.74%	1.39%	*p* = 0.830
	2 Fractures		24.79%	6.41%	7.52%	1.67%	
	3 Fractures		16.43%	4.56%	5.01%	0.28%	
	4 Fractures		1.39%	0.56%	0%	0%	
Month of injury	January	395	**4.56%**	**1.01%**	**0%**	**0.25%**	***p*** **= 0.015**
	February		**0.76%**	**0.25%**	**0.25%**	**0%**	
	March		**4.30%**	**0.51%**	**0.25%**	**0%**	
	April		**3.54%**	**0.76%**	**2.78%**	**0%**	
	May		**5.32%**	**2.53%**	**1.77%**	**0.51%**	
	June		**5.32%**	**2.78%**	**1.01%**	**0.51%**	
	July		**8.35%**	**0.76%**	**3.29%**	**0.51%**	
	August		**9.87%**	**1.01%**	**3.54%**	**0.25%**	
	September		**7.34%**	**2.53%**	**1.01%**	**0.51%**	
	October		**5.23%**	**0.51%**	**2.03%**	**0.25%**	
	November		**4.30%**	**0.51%**	**0.76%**	**0.25%**	
	December		**4.30%**	**3.04%**	**0.25%**	**0%**	
Place of first treatment	Another center	395	4.56%	2.53%	1.01%	0.25%	*p* = 0.240
	Own patient		58.23%	14.18%	16.20%	3.04%	
Delay of Surgery [days]	Full cohort	395	8.24 ± 7.03	8.07 ± 5.12	9.79 ± 11.66	24.42 ± 50.96	*p* = 0.898
Duration of Surgery [minutes]	Full cohort	395	187.24 ± 83.38	196.85 ± 87.15	195.25 ± 82.20	204.09 ± 90.47	*p* = 0.702
Surgical approach	Auricular	359	0.84%	0%	0%	0%	*p* = 0.424
	Extended preauricular		17.27%	4.46%	5.57%	1.39%	
	Extended retromandibular		5.85%	1.95%	3.34%	0.28%	
	Intraoral		2.23%	1.95%	0.28%	0.28%	
	Periangular		1.67%	0.28%	0.28%	0%	
	Preauricular		19.22%	5.01%	3.62%	0.56%	
	Retroauricular		0.84%	0.56%	0.25%	0%	
	Retromandibular		14.76%	2.51%	3.90%	0.84%	
Comorbidity	Generally healthy	355	46.20%	9.01%	10.99%	1.41%	*p* = 0.175
	1 Disease		12.39%	5.07%	4.79%	1.13%	
	2 Diseases		2.54%	1.97%	0.85%	0.28%	
	3 Diseases		1.69%	0.85%	0.28%	0%	
	4 Diseases		0.28%	0%	0%	0%	
	5 Diseases		0%	0%	0.28%	0%	
Pre-operative HGB [g/dL]	Full cohort	395	14.14 ± 1.61	14.13 ± 1.54	13.99 ± 1.98	14.10 ± 1.68	*p* = 0.926
Pre-operative WBC [×10^9^/L]	Full cohort	395	8.01 ± 3.29	7.86 ± 3.03	8.48 ± 3.18	8.11 ± 2.02	*p* = 0.488
Screw number for CHF	1 Screw	139	1.44%	2.88%	1.44%	0%	*p* = 0.174
	2 Screws		43.32%	12.23%	12.95%	2.16%	
	3 Screws		11.51%	3.60%	2.88%	1.44%	
	4 Screws		0.72%	0%	1.44%	0%	
Screw material for CHF	Magnesium	139	5.76%	0.72%	2.88%	0%	*p* = 0.461
	Titanium		53.82%	17.99%	15.83%	3.60%	
Fixing material	Compressive screws	355	22.82%	7.61%	7.04%	1.41%	*p* = 0.661
	XCP plate		3.66%	0.85%	0.28%	0.56%	
	ACP plate		22.78%	4.23%	6.20%	1.13%	
	3 Straight plates		1.13%	0.28%	0.28%	0%	
	2 Straight plates		12.11%	3.10%	3.10%	0.28%	
	1 Straight plate		0.28%	0.56%	0.28%	0%	
Salivary fistula	Yes	359	5.57%	1.11%	1.39%	0.28%	*p* = 0.957
	No		57.10%	15.60%	15.88%	3.06%	
Periauricular skin desensitization	Yes	359	11.14%	4.18%	3.62%	0.56%	*p* = 0.631
	No		51.53%	12.53%	13.65%	2.79%	
Facial nerve intraoperational neurotmesis	Yes	359	1.39%	0.28%	0.28%	0%	*p* = 0.943
	No		61.28%	16.43%	16.99%	3.34%	

Values are presented as mean ± standard deviation (SD) for continuous variables and n (%) for categorical variables. Available N indicates the number of cases for each variable; denominators may differ across variables. The variables “Screw number for CHF” and “Screw material for CHF” have full N = 139, as they apply exclusively to patients with condylar head fractures. Abbreviations: H0, H1, H2, H3—successive grades of the Helkimo Dysfunction Index grades; CHF—Condylar Head Fracture. Statistically, significant values (*p* < 0.05) are indicated in bold.
